# The Identification and Structure of an N-Terminal PR Domain Show that FOG1 Is a Member of the PRDM Family of Proteins

**DOI:** 10.1371/journal.pone.0106011

**Published:** 2014-08-27

**Authors:** Molly K. Clifton, Belinda J. Westman, Sock Yue Thong, Mitchell R. O’Connell, Michael W. Webster, Nicholas E. Shepherd, Kate G. Quinlan, Merlin Crossley, Gerd A. Blobel, Joel P. Mackay

**Affiliations:** 1 School of Molecular Bioscience, University of Sydney, Sydney, NSW, Australia; 2 Division of Hematology, The Children's Hospital of Philadelphia, The Perelman School of Medicine at the University of Pennsylvania, Philadelphia, Pennsylvania, United States of America; Institute of Enzymology of the Hungarian Academy of Science, Hungary

## Abstract

FOG1 is a transcriptional regulator that acts in concert with the hematopoietic master regulator GATA1 to coordinate the differentiation of platelets and erythrocytes. Despite considerable effort, however, the mechanisms through which FOG1 regulates gene expression are only partially understood. Here we report the discovery of a previously unrecognized domain in FOG1: a PR (PRD-BF1 and RIZ) domain that is distantly related in sequence to the SET domains that are found in many histone methyltransferases. We have used NMR spectroscopy to determine the solution structure of this domain, revealing that the domain shares close structural similarity with SET domains. Titration with *S*-adenosyl-L-homocysteine, the cofactor product synonymous with SET domain methyltransferase activity, indicated that the FOG PR domain is not, however, likely to function as a methyltransferase in the same fashion. We also sought to define the function of this domain using both pulldown experiments and gel shift assays. However, neither pulldowns from mammalian nuclear extracts nor yeast two-hybrid assays reproducibly revealed binding partners, and we were unable to detect nucleic-acid-binding activity in this domain using our high-diversity Pentaprobe oligonucleotides. Overall, our data demonstrate that FOG1 is a member of the PRDM (PR domain containing proteins, with zinc fingers) family of transcriptional regulators. The function of many PR domains, however, remains somewhat enigmatic for the time being.

## Introduction

**Figure 1:**
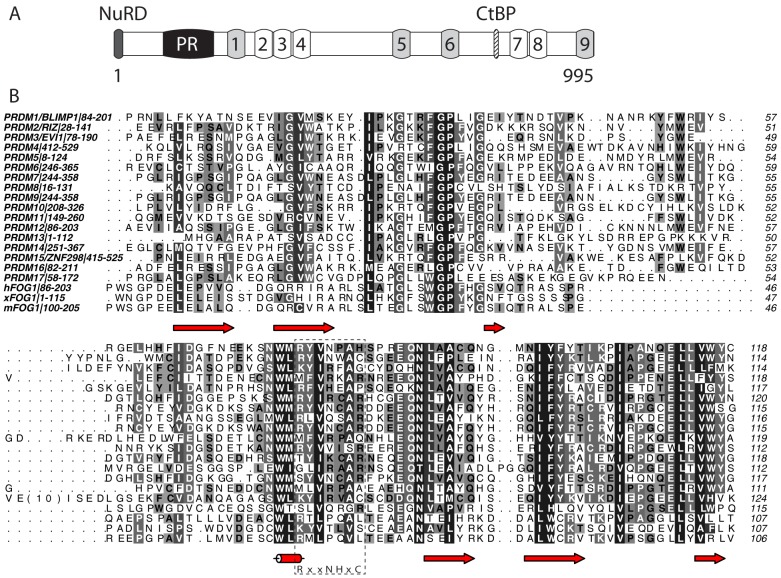
Domain structure and sequence analysis of FOG1. (A) Domain structure of murine FOG1. C2HC type zinc fingers are *light grey*; C2H2 fingers are unshaded. The binding sites for CtBP and the NuRD complex are indicated, as is the newly identified PR domain. (B) Sequence alignment of murine FOG(100–205) with human and *Xenopus laevis* FOG1 and with all human PR domains. Colouring indicates conservation at four different levels. The essential catalytic consensus motif found in SET domains is shown below the alignment and indicated with a dashed box. Secondary structure elements in FOG-PR are indicated below the alignment. Alignment was carried out using CLUSTAL OMEGA [49] and the diagram made using ALINE [50].

The activity of the transcription factor GATA1 in erythroid development is modulated by a range of coregulators, including Friend of GATA 1 (FOG1). FOG1 is a nine-zinc-finger protein **(** **Figure 1A** **)** that is essential for proper differentiation and maturation of both megakaryocytes and erythroid precursors [1]. FOG1 knockout mice die at E10.5–11.5 due to severe anaemia with arrest in erythroid development, a phenotype that is related to that observed in GATA1 knockout mice [2]. FOG1 and GATA1 interact both functionally [3] and physically [4], and disruption of the normal interaction of FOG1 and GATA1 has been linked to a range of inherited blood disorders (reviewed in [5]).

Despite FOG1 containing nine classical zinc-finger domains, there is no evidence to date that the protein binds directly to nucleic acids, suggesting that FOG1 most likely regulates GATA1 activity by recruiting co-regulator complexes. FOG1 is required for both the activation and the repression of most GATA1 target genes [6]–[9]. FOG-mediated repression of GATA1 in transient transfection assays and ectopic expression both depend on its ability to recruit the co-repressor C-terminal binding protein (CtBP) [10]–[12], via a PXDL motif between zinc fingers 6 and 7. However, FOG1 does not appear to require its major PXDLS CtBP-binding motif during erythropoiesis since mice carrying a FOG1 mutant with reduced CtBP binding develop normally [13], suggesting FOG1 recruits another repressor complex during erythropoiesis.

The N-terminus of FOG1 appears to be particularly important for its function. During megakaryopoiesis, deletion mutants lacking residues 1–144 could at least partially rescue erythroid but not megakaryocyte maturation, suggesting a lineage specific role for the N-terminal region [13]. Subsequently, residues 1–12 of FOG1 were shown to be able to mediate transcriptional repression by GATA1 [14], via recruitment of the nucleosome remodeling and deacetylation (NuRD) complex [15]–[17]. Similarly, the N-terminal region of FOG2 represses GATA-4 activity [17], although the possibility remains open that other regions might contribute to repression.

As part of an effort to understand the molecular mechanisms through which FOG1 regulates gene expression during hematopoietic development, we analyzed the amino acid sequence of murine FOG1 (Uniprot: O35615). PONDR (http://www.pondr.com/), a program that predicts the distribution of structured and natively disordered regions in a protein sequence, predicted that part of the region P100–V254 of FOG1 is likely to be well-ordered. Sequence comparisons reveal similarity of up to ∼30% to the PR (PRDI-BF1 and RIZ homology) domains found in the human proteins PRDM1–17 **(** **Figure 1B** **)**. The relatively low degree of similarity means that programs such as Interpro (http://www.ebi.ac.uk/interpro/) do not reveal any domains in FOG1 other than the nine well-characterized classical zinc fingers indicated in **Figure 1A**.

PRDM-family proteins are gene regulatory proteins that are found in metazoans, but not plants or fungi. Seventeen such proteins have been defined in primates, whereas only two are found in the sea squirt *Ciona intestinalis*, indicating a substantial expansion during vertebrate evolution. Their biological roles are still not well understood in many cases, but a number of family members appear to act in stem cells and in cellular differentiation (reviewed in [18]). PRDM14 is important in stem cell biology and epigenetic reprogramming (reviewed in [19]), PRDM3 is required for the integrity of heterochromatin [20] and PRDM16 is essential for maintaining adipocyte identity [21]. Not surprisingly therefore, dysregulation of PRDM activity has been associated with several different types of cancer [22]–[24].

All 17 human proteins contain an N-terminal PR domain and all but PRDM11 contain an array of between four and fifteen classical zinc fingers clustered in a range of different patterns [18], [25]. The PR domain bears structural similarity to the catalytic SET domains (named for the *Drosophila* proteins Suppressor of variegation 3–9, Enhancer of zeste and Trithorax) found in histone lysine methyltransferases [26], although in general many of the residues associated with catalytic activity in the SET domains are not conserved in PR domains. Despite the absence of these residues, however, methyltransferase activity *has* been observed in at a number of PRDM proteins (PRDM2, -3, -6, -8, -9 and -16) [20], [27]–[31]. Members of the family have also been demonstrated to act as sequence-specific DNA-binding proteins (most likely through their zinc-finger domains) or as protein-recruitment agents at gene regulatory elements, and at this stage a clear consensus view of the function of these proteins as a class has not yet emerged.

### Determination of the solution structure of FOG1-PR

**Figure 2:**
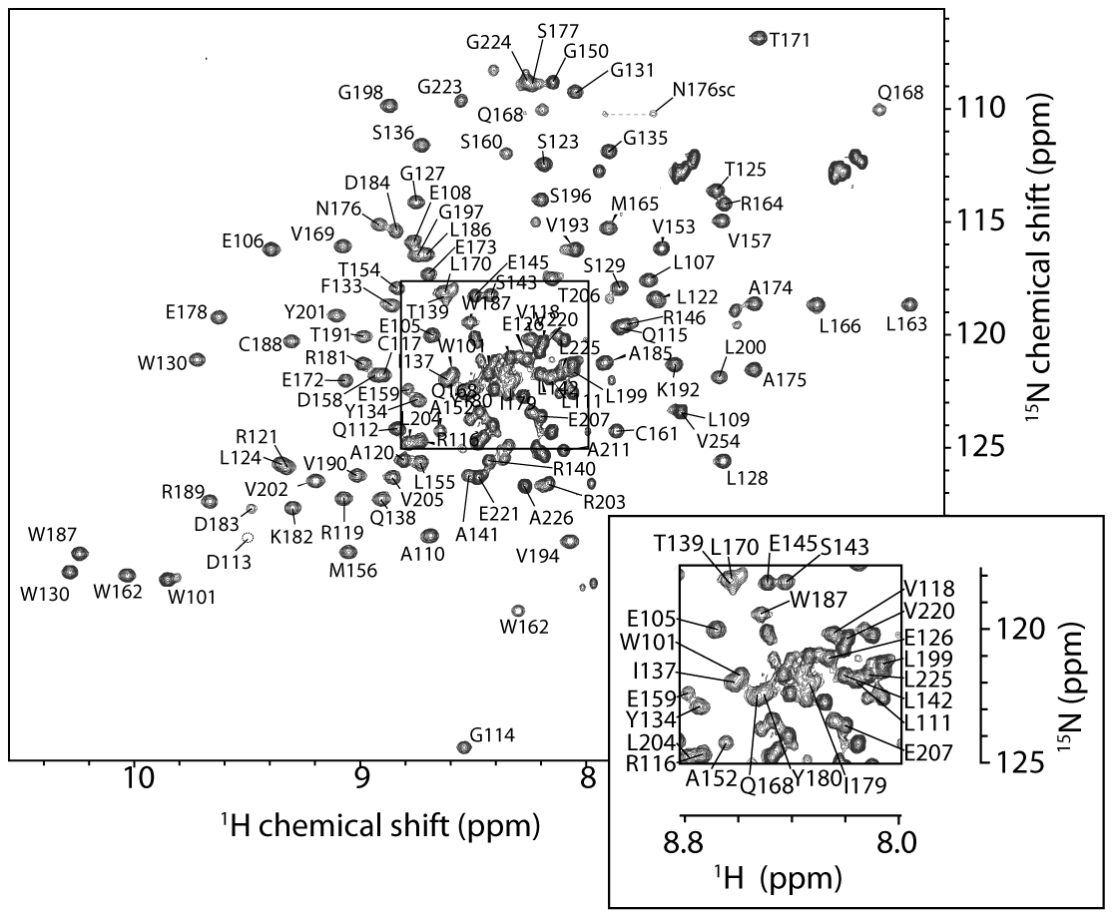
^15^N-HSQC spectrum of FOG1. The spectrum was recorded on 0.5 mM FOG-PR at 298 K on a 600-MHz NMR spectrometer. The central portion of the spectrum (boxed) is labeled with assignments in the expansion. Signals with no label are unassigned.

To characterize this predicted domain in FOG1, we overexpressed (as a GST-fusion protein in *Escherichia coli*) a polypeptide corresponding to residues P100–V254 of murine FOG1 (hereafter referred to as FOG-PR) and then purified it using GSH-affinity and, following removal of the GST by cleavage with thrombin, size exclusion chromatography. Far-UV circular dichroism and one-dimensional ^1^H NMR spectra (not shown) revealed that this polypeptide contained substantial β-sheet secondary structure and took up a well-defined conformation in solution. Size exclusion chromatography with in-line multi-angle laser light scattering (MALLS) gave a mass estimate of ∼17.2 kDa, indicating that FOG-PR (MW_theor._ = 17.0 kDa) is monomeric in solution and suggesting that FOG-PR should be a suitable candidate for structural analysis by NMR spectroscopy. Accordingly, the ^15^N-HSQC spectrum of FOG-PR contains approximately the expected number of signals for a 150-residue protein and displays linewidths and chemical shift dispersion consistent with a folded monomer (**Figure 2**).

We went on to determine the solution structure of FOG-PR using multidimensional heteronuclear NMR methods. Assignments were made for ∼95% of expected backbone ^1^H, ^13^C and ^15^N nuclei, and ∼85% of side chain ^1^H, ^13^C nuclei in the region P100–E207. Little or no data were observed for the residues E147 and E148, and assignments could only be made with confidence for a small number of residues in the C-terminal region (P208–V254). Approximately 25 signals in the ^15^N-HSQC therefore remained unassigned, but nearly all of these had rather narrow linewidths, H^N^ chemical shifts in the range ∼7.8–8.5 ppm and few NOEs in a ^15^N-edited NOESY spectrum. Taken together, these observations indicate that the C-terminal part of the polypeptide is disordered. The results of limited proteolysis carried out on FOG1(100–254) were also consistent with this conclusion, identifying a major proteolysis product corresponding to FOG1(100–214).

**Figure 3:**
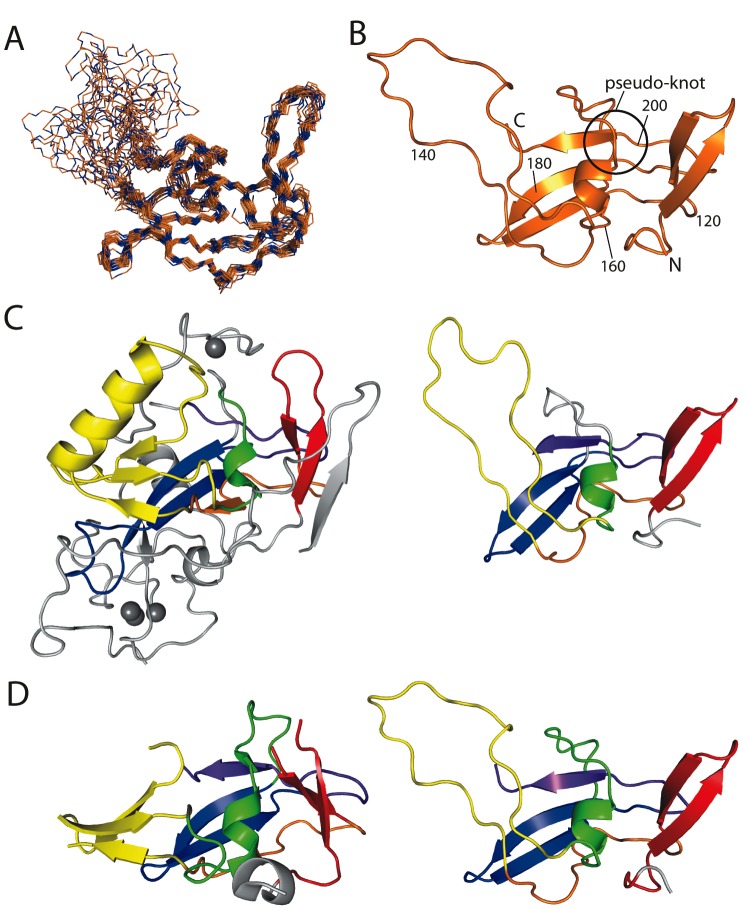
NMR solution structure of FOG-PR. (a) Overlay of backbone traces of the 20 lowest energy structures following RECOORD refinement. NMR data indicated that residues 100–102 and 207–254 are disordered, and these are not shown. (b) Ribbon diagram of the lowest energy structure of FOG-PR. The N/C-termini, residue numbers and the position of the pseudoknot are indicated. (c) Comparison of the DIM5 SET domain structure (PDB: 1PEG [36], *left*) with FOG-PR (*right*). Corresponding elements of structure are coloured similarly in a pattern ranging from red to orange to yellow to green to blue to purple (N- to C-terminal end). The regions in gray are a small pre-SET domain and the SET-I variable region that lies between the yellow and green regions. The pseudoknot is apparent as the purple C-terminal β-strand that passes through the green helix/loop. Zinc ions are shown as grey spheres. (d) Comparison of the PRDM4 PR domain structure (*left*, PDB 3DB5) with FOG-PR (*right*). Corresponding elements of structure are coloured as in part (b). Diagrams were produced using the programs PYMOL and MOLMOL.

**Figure 4:**
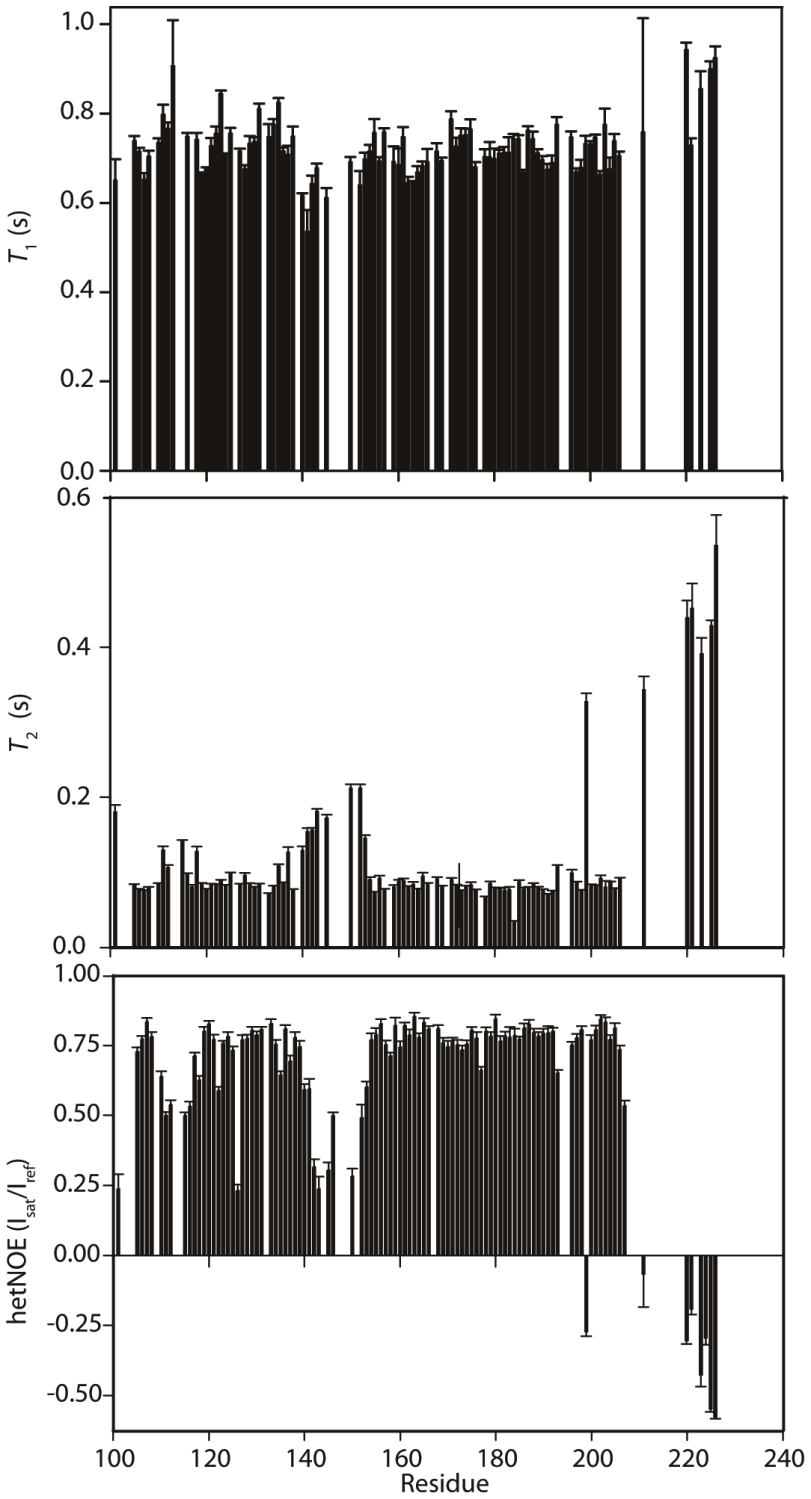
^15^N relaxation data for FOG-PR. Backbone ^15^N *T*_1_, *T*_2_ and heteronuclear ^15^N-^1^H NOE values are plotted as a function of residue number. Error bars indicate the standard error from the curve fit for *T*_1_ and *T*_2_ values and the range taken from duplicate measurements for the ^15^N-^1^H NOE.

^15^N-edited, ^13^C-edited and 2D NOESY spectra were peak-picked and CYANA 3.0 [32] was used to assign NOEs and calculate structures. The 50 lowest energy structures, calculated with 1244 distance restraints and TALOS+-derived dihedral angle restraints for 89 residues, were refined in explicit water using CNS, according to the RECOORD protocol [33]. The 20 lowest energy structures were used to represent the structure of FOG-PR (**Figure 3** and **Table 1**). This family of structures has a backbone RMSD (over residues with φ and ψ angle order parameters of >0.95) of 0.67 Å, and exhibits no NOE violations >0.5 Å. The well-ordered region of the protein was defined as ranging from G103–I137 and D158–V205; the sequence Q138–V157 and the sequences N- and C-terminal to the ordered region exhibited random-coil chemical shifts, gave rise to no non-sequential NOEs, and had very low backbone angle order parameters in the final structures. PROCHECK_NMR analysis showed that, on a Ramachandran plot, 99.8% of residues in the well-ordered region of the protein fall within the most favoured or additionally allowed regions (calculated for non-Pro, non-Gly residues). ^15^N *T*_1_, *T*_2_ and heteronuclear NOE data were consistent with this arrangement (**Figure 4**).

**Table 1:** Experimental restraints and structural statistics for FOG-PR.

*Experimental restraints*	
Distance restraints	1230
Intraresidue (*i,i*)	289
Sequential (*i,i*+1)	366
Medium-range (2≤ |*i*–*j*| ≤4)	124
Long-range (|*i*–*j*| >4)	451
Total dihedral angle restraints	
φ	89
ψ	89
*Pairwise RMS deviation from mean structure (residues 103–137, 158–205)*	
Backbone atoms (N, C^α^, C)	0.67±0.13 Å
All heavy atoms (N, C, O, S)	1.13±0.15
*PROCHECK statistics*	
Residues in most favoured region	92.4%
Residues in additional allowed regions	7.4%
Residues in generously allowed regions	0.2%
Residues in disallowed regions	0%
*Deviation from idealized geometry (residues 100–226)*	
Bond lengths	0.017 Å
Bond angles	1.4°

### Comparison of the structure with SET and other PR domains

The region encompassing the PR domain (P100–V205) of murine FOG1 has ∼90% and ∼67% sequence similarity with the corresponding regions of human FOG1 and *Xenopus laevis* FOG1, respectively, indicating that this structure is conserved across all FOG1 homologues. It is also clearly conserved in FOG2, although does not appear to be present in the related *Drosophila* protein U-shaped. Overall, the fold closely resembles that of the enzymatic SET domains found in many lysine methyltransferases [34]. Examination of the >10 structures of SET domains determined to date shows that the domain consists of a number of semi-modular subunits. The core SET domain appears to comprise N- and C-terminal regions (SET-N and SET-C) that are relatively invariant between different domains, together with a central SET-I region that has widely varying length and structure [35]. In addition, flanking domains (pre-SET and post-SET domains) are generally observed, which are also somewhat variable in nature.

**Figure 3C** shows a comparison of the structure of FOG-PR and the core of the SET domain of DIM-5 [36]. The arrangement of secondary structural elements is clearly the same, down to the presence of the C-terminal pseudo-knot that is common to all SET domain structures solved to date (purple). The SET-N and SET-C regions clearly match those in DIM-5. In contrast, SET-I is an α+β structure in DIM-5 (yellow), whereas in FOG this region simply comprises a disordered 12-residue loop. The residues in this loop gave rise to broad (or no) signals in the NMR spectra, indicating that they participate in µs-ms timescale motion; it is possible that this region forms a marginally stable structure. DIM-5 also displays an elaborate pre-SET domain (grey) that binds three Zn(II) ions. A structure-guided alignment of the DIM-5 and FOG-PR sequences reveals that the most highly clustered set of conserved residues lies in the hydrophobic core that is common to the two structures.

Structures have also been determined for the PR domains of PRDM1, 2, 4, 9, 10, 11 and 12 (although only structures of PRDM2 have been published [37], [38]). These structures more closely resemble that of FOG-PR (**Figure 3D** shows a comparison of FOG-PR with the PRDM4 PR domain), with fewer elaborations than the SET domains. Notably, all seven structures of PR domains display a three-stranded β-sheet in the SET-I region that is disordered in FOG-PR, although some poorly defined electron density is observed in several PR structures, perhaps indicating the presence of some flexibility in this region.

### FOG-PR is unlikely to have methyltransferase activity

SET domains possess histone methyltransferase activity towards specific lysine residues in histones tails, leading to positive and negative regulation of gene expression, depending on context. The transferred methyl group is derived from the cofactor *S*-adenyosyl-L-methionine (SAM), and the transfer reaction gives rise to the cofactor product *S*-adenyosyl-L-homocysteine (AdoHcy). Several structures have been determined of SET domains in the presence of AdoHcy, allowing the identification of the conserved substrate-binding pocket of the protein (see, for example, [39], [40]). To test whether or not FOG-PR might also act as a methyltransferase domain, we titrated AdoHcy into a solution of ^15^N-labeled FOG-PR and recorded ^15^N-HSQC spectra. No changes were observed following the addition of up to 100 molar equivalents of AdoHcy (not shown), suggesting that FOG-PR is unlikely to act as a SAM dependent methyltransferase.

This lack of binding is consistent with the absence of asparagine and cysteine residues that are highly conserved in the Ado-Met/AdoHcy co-factor binding region of SET domains [35], [40]. These residues form part of an H/RxxNHxC motif that is thought to be important for enzymatic activity. As noted above, methyltransferase activity has been observed in at a number of PRDM proteins, suggesting that other residues might well be able to fulfil their roles. In the case of PRDM9, a structure has been determined of the PR domain bound to both a histone H3-derived substrate peptide and AdoHcy [41]. It is notable that a substantial portion of the binding site for both molecules is derived from residues in either the SET-I region or in the sequence immediately C-terminal to the pseudo-knot. Both of these regions are disordered in the FOG-PR structure and in general the residues that make contacts with AdoHcy and histone H3 do not appear to be conserved in FOG-PR.

### Further efforts to pinpoint the function of FOG-PR

If only some PR domains act as methyltransferases, the question arises as to what the function is of the remaining domains. Some PRDM proteins have been shown to associate with DNA and to recruit other proteins to chromatin [18], and it is therefore possible that PR domains can act as either DNA- or protein-binding modules. We used gel shift assays to assess the DNA-binding properties of FOG-PR. Previously we described Pentaprobes, a set of six high-diversity oligonucleotides that contain all possible five-base-pair sequences [42]. Data from our lab indicate that bona fide DNA-binding proteins will typically bind to Pentaprobes in a gel shift assay. However, we observed no binding of FOG-PR to any of the six double-stranded Pentaprobes. Similarly, no binding was observed to single-stranded forms of the Pentaprobes (data not shown).

The PR domain of PRDM2 (RIZ) has been shown in GST-pulldowns to act as a protein interaction domain, mediating homodimerization [43]. We tested the protein-binding capability of FOG-PR by binding GST-FOG-PR to glutathione agarose beads and treating the beads with a nuclear extract from murine erythroleukemia (MEL) cells. SDS-PAGE analysis did not reveal any bands of significant intensity that were not observed in a GST-only control pulldown (data not shown). Furthermore, yeast two-hybrid screens carried out using FOG-PR as a bait and cDNA libraries from murine erythroleukemia (MEL) or K262 cells as prey did not yield any high-confidence hits (data not shown).

### Implications for PR domain function

Although it is well accepted that the PR family of proteins acts to regulate gene expression, there is not yet a clear consensus on the biochemical mechanisms through which they achieve this outcome. Only a subset of the proteins have been demonstrated to display methyltransferase activity and, given the lack of clearly identified catalytic residues, it is possible that the observed activity arises (at least in some cases) from co-purified proteins. PR domains lacking catalytic activity might still function as interaction modules that recognize methylated histone tails or methylated sequences from other proteins. Such a binding activity could serve to modulate their function by either influencing their targeting to specific genomic loci or by regulating their binding to other protein partners that can be ‘tagged’ by lysine methylation. It is notable that a catalytically inactive SET domain has also been observed in the human protein SUVH9 [44]; the function of this domain is also currently unresolved.

DNA-binding activity has been reported for several family members, consistent with the presence of multiple classical zinc finger domains (reviewed in [18]). In contrast, the zinc fingers of FOG1 do not appear to bind DNA (unpublished data). It is, however, known that zinc fingers in FOG1 act as protein recruitment domains, binding to GATA1 [3], [4] and to TACC3 [45] and it is possible that a subset of the zinc fingers in PRDM-family proteins (especially zinc fingers that are not part of a tandem repeat) likewise act to recognize protein partners.

Other protein-recruitment motifs exist in PRDM-family proteins, including KRAB and AWS repressor domains. FOG1 also harbors several domains that are associated with recruiting corepressors, including an N-terminal sequence that recruits the Nucleosome Remodeling and Deacetylase (NuRD) complex to chromatin and a C-terminal Binding Protein (CtBP) binding motif [12] (Figure 1). Notably, PRDM2, 3 and 16 also contain CtBP binding motifs.

In summary, our data show that FOG1 contains a PR domain. The presence of this domain, together with other structural and functional similarities, defines FOG1 as a new member of the PR-domain-containing protein family. This family of transcriptional regulators is likely to share a common mechanism of action and a broader elucidation of the biochemical function of the PR domain will illuminate the activity of the whole family.

## Materials and Methods

### Protein production

GST-mFOG1(100−254) (FOG-PR) in pGEX-2T was overexpressed in the BL21 strain of *Escherichia coli* (0.4 mM IPTG, 37°C). For overexpression of ^15^N- or ^15^N,^13^C-labeled FOG-PR, the protocol of Cai *et al.* [46] was used, with induction occurring overnight at 25°C. Cell pellets were lysed by sonication (in 50 mM Tris pH 8.0, 50 mM NaCl, 1% Triton X-100, 1 mM PMSF, 1 mM ß-mercaptoethanol, 0.1 mg/mL lysozyme), clarified by centrifugation and the supernatant bound to a glutathione Sepharose column at 4°C for 1–2 h. After washing (50 mM Tris, 150 mM NaCl, 2.5 mM CaCl_2_), the fusion protein was either eluted with glutathione or cleaved with thrombin (37°C, 2–3 h). The eluted and concentrated protein (3-kDa cutoff Centricon) was further purified on a Superdex 200 16/60 or Superose 12 HR 10/30 column (GE) in 50 mM sodium phosphate, 150 mM NaCl, 1 mM DTT, pH 7.2. For NMR measurements, FOG(100–254) was prepared at 0.5–1.0 mM in a solution containing 20 mM Na_2_HPO_4_/NaH_2_PO_4_, pH 7.0 (5% ^2^H_2_O) and 2 µM 5,5-dimethylsilapentanesulfonate (DSS).

### NMR spectroscopy and structure calculations

Resonance assignments were made using a standard set of triple resonance experiments and NOE data were obtained from ^13^C-NOESY-HSQC (in >99% ^2^H_2_O) and ^15^N-NOESY-HSQC spectra. All NMR spectra were recorded at 298 K on a Bruker Avance 600 MHz spectrometers, processed using TOPSPIN and analyzed using Sparky (T. D. Goddard and D. G. Kneller, SPARKY 3, University of California, San Francisco). ^15^N backbone relaxation experiments (*T*_1_, *T*_2_ and heteronuclear NOE) were performed using standard Bruker pulse programs and were analyzed to extract relaxation rates using Sparky. Backbone φ and ψ dihedral angle restraints were derived from the assigned backbone chemical shifts using TALOS+ [47]. Automated NOE assignment and structure calculations were carried out using CYANA [32] and the lowest energy structures were refined using the RECOORD protocol [33]. The 20 conformers with the lowest energy were used to represent the solution structure of FOG-PR and deposited in the Protein Data Bank (PDB accession number 2mpl). Geometrical properties were assessed using PROCHECK_NMR [48].

### Limited proteolysis

Limited proteolysis was carried out by treating 100 µg FOG1(100–254) with 1 µg of chymotrypsin in a 20-µL reaction volume for 4–10 min, separating the reaction mixture by SDS-PAGE and analyzing the major bands by peptide mass fingerprinting on a MALDI mass spectrometer.

### Titration with AdoHcy

AdoHcy in a matched buffer was added to ^15^N-labeled FOG-PR [in 20 mM Na_2_HPO_4_/NaH_2_PO_4_, pH 7.0 (5% ^2^H_2_O) and 2 µM DSS] giving final concentrations of up to 20 mM AdoHcy. ^15^N-HSQC spectra were recorded as above.

### Gel shift assays

The double-stranded probes were end-labeled with ^32^P according to standard procedures using polynucleotide kinase and purified on native polyacrylamide gels by standard methods [42]. Gel shift reactions were set up in a total volume of 30 µl, comprising approximately 1 pmol of ^32^P labeled probe, ∼100 ng of recombinant protein, 10 mM Hepes, pH 7.8, 50 mM KCl, 5 mM MgCl_2_, 1 mM EDTA and 5% glycerol. After incubation on ice for 10 min, the samples were loaded onto a 6% native polyacrylamide gel made up in 0.5× TBE. The gel was then subjected to electrophoresis at 15 V/cm and 4°C for 3 h, dried, analysed and quantified when necessary using a PhosphoImager (Molecular Dynamics).

### Pulldown assay

Mouse erythroleukemia (MEL) cells were cultured in DMEM medium (+glucose, +glutamine, +pyruvate) supplemented with 5% FBS and 1% penicillin/streptomycin. 10–20 mL seed cultures were maintained in T75 flasks. For 1 L grow-ups, 1 mL of seed culture was added to 250 ml fresh medium in a T175 flask and grown at 37°C, 5% CO_2_ for 72 h to a density of ∼1×10^6^ cells/mL (viability >85%). Cells were harvested by centrifugation at 2000 rpm for 5 min to yield 1–1.5 g (wet weight) cells/L culture. Cells were washed twice with PBS, then swollen in hypotonic solution (10 mM HEPES, 1.5 mM MgCl_2_, 10 mM KCl, pH 7.9) for 20 min and frozen in liquid nitrogen and stored at –80°C until use. Frozen and swollen cells were thawed at 37°C for 10 min and treated with IGEPAL (0.6% v/v) for 10 min. The mixture was centrifuged for 5 min at 2000 rpm to pellet nuclei and the cytoplasmic supernatant was discarded. The pellet was gently washed once with hypotonic solution (10 mM HEPES, 1.5 mM MgCl_2_, 10 mM KCl, pH 7.9, 0.6% IGEPAL) and centrifuged again at 2000 rpm. Next buffer A (50 mM Tris, 150 mM NaCl, 1% Triton X-100, 1 mM DTT, Complete protease inhibitors, pH 7.4) was added to the pellet (3 ml/g cells) and the mixture was sonicated on ice (step-tip, 10×1 s bursts with 10 s recovery, three times total) to give a milky white solution. The mixture was centrifuged at 13000 rpm for 10 min at 4°C. The clear nuclear extract was then used immediately.

For the pulldown, GST-FOG-PR GSH beads (200 µL beads, 0.5 mL beads per liter of *E. coli* lysate) was incubated with MEL nuclear extract (from 0.5 L MEL cell culture) overnight at 4°C. The beads were separated from the nuclear extract and washed three times with buffer A. Gel loading dye (LDS, 10 µL) was added to the wet beads and the sample heated for 10 min at 90°C. The mixture was then analysed by SDS PAGE.

### Protein Data Bank accession codes

^1^H, ^13^C and ^15^N backbone and sidechain chemical shift assignments have been deposited in the BioMagResBank with accession number 19988 and the structure coordinates have been deposited in the RCSB Protein Data Bank (accession code 2 mpl).

## References

[1] Tsang AP, Fujiwara Y, Hom DB, Orkin SH (1998) Failure of megakaryopoiesis and arrested erythropoiesis in mice lacking the GATA-1 transcriptional cofactor FOG. Genes Dev 12: 1176–1188. 9553047 10.1101/gad.12.8.1176 PMC316724

[2] Cantor AB, Orkin SH (2002b) Transcriptional regulation of erythropoiesis: an affair involving multiple partners. Oncogene 21: 3368–3376. 12032775 10.1038/sj.onc.1205326

[3] Tsang AP, Visvader JE, Turner CA, Fujiwara Y, Yu C, et al (1997) FOG, a multitype zinc finger protein, acts as a cofactor for transcription factor GATA-1 in erythroid and megakaryocytic differentiation. Cell 90: 109–119. 9230307 10.1016/s0092-8674(00)80318-9

[4] Liew CK, Simpson RJ, Kwan AH, Crofts LA, Loughlin FE, et al (2005) Zinc fingers as protein recognition motifs: structural basis for the GATA-1/friend of GATA interaction. Proceedings of the National Academy of Sciences of the United States of America 102: 583–588. 15644435 10.1073/pnas.0407511102 PMC545545

[5] Ciovacco WA, Raskind WH, Kacena MA (2008) Human phenotypes associated with GATA-1 mutations. Gene 427: 1–6. 18930124 10.1016/j.gene.2008.09.018 PMC2601579

[6] Muntean AG, Crispino JD (2005) Differential requirements for the activation domain and FOG-interaction surface of GATA-1 in megakaryocyte gene expression and development. Blood 106: 1223–1231. 15860665 10.1182/blood-2005-02-0551 PMC1895209

[7] Crispino JD, Lodish MB, MacKay JP, Orkin SH (1999) Use of altered specificity mutants to probe a specific protein-protein interaction in differentiation: the GATA-1:FOG complex. Molecular cell 3: 219–228. 10078204 10.1016/s1097-2765(00)80312-3

[8] Pal S, Cantor AB, Johnson KD, Moran TB, Boyer ME, et al (2004) Coregulator-dependent facilitation of chromatin occupancy by GATA-1. Proceedings of the National Academy of Sciences of the United States of America 101: 980–985. 14715908 10.1073/pnas.0307612100 PMC327128

[9] Wang X, Crispino JD, Letting DL, Nakazawa M, Poncz M, et al (2002) Control of megakaryocyte-specific gene expression by GATA-1 and FOG-1: role of Ets transcription factors. The EMBO journal 21: 5225–5234. 12356738 10.1093/emboj/cdf527 PMC129049

[10] Deconinck AE, Mead PE, Tevosian SG, Crispino JD, Katz SG, et al (2000) FOG acts as a repressor of red blood cell development in Xenopus. Development 127: 2031–2040. 10769228 10.1242/dev.127.10.2031

[11] Fossett N, Tevosian SG, Gajewski K, Zhang Q, Orkin SH, et al (2001) The Friend of GATA proteins U-shaped, FOG-1, and FOG-2 function as negative regulators of blood, heart, and eye development in Drosophila. Proc Natl Acad Sci U S A 98: 7342–7347. 11404479 10.1073/pnas.131215798 PMC34670

[12] Turner J, Crossley M (1998) Cloning and characterization of mCtBP2, a co-repressor that associates with basic Kruppel-like factor and other mammalian transcriptional regulators. The EMBO journal 17: 5129–5140. 9724649 10.1093/emboj/17.17.5129 PMC1170841

[13] Cantor AB, Katz SG, Orkin SH (2002a) Distinct domains of the GATA-1 cofactor FOG-1 differentially influence erythroid versus megakaryocytic maturation. Mol Cell Biol 22: 4268–4279. 12024038 10.1128/MCB.22.12.4268-4279.2002 PMC133877

[14] Lin AC, Roche AE, Wilk J, Svensson EC (2004) The N termini of Friend of GATA (FOG) proteins define a novel transcriptional repression motif and a superfamily of transcriptional repressors. J Biol Chem 279: 55017–55023. 15507435 10.1074/jbc.M411240200

[15] Hong W, Nakazawa M, Chen YY, Kori R, Vakoc CR, et al (2005) FOG-1 recruits the NuRD repressor complex to mediate transcriptional repression by GATA-1. Embo J 24: 2367–2378. 15920470 10.1038/sj.emboj.7600703 PMC1173144

[16] Lejon S, Thong SY, Murthy A, AlQarni S, Murzina NV, et al (2011) Insights into association of the NuRD complex with FOG-1 from the crystal structure of an RbAp48.FOG-1 complex. The Journal of biological chemistry 286: 1196–1203. 21047798 10.1074/jbc.M110.195842 PMC3020727

[17] Svensson EC, Huggins GS, Dardik FB, Polk CE, Leiden JM (2000) A functionally conserved N-terminal domain of the friend of GATA-2 (FOG-2) protein represses GATA4-dependent transcription. J Biol Chem 275: 20762–20769. 10801815 10.1074/jbc.M001522200

[18] Hohenauer T, Moore AW (2012) The Prdm family: expanding roles in stem cells and development. Development 139: 2267–2282. 22669819 10.1242/dev.070110

[19] Nakaki F, Saitou M (2014) PRDM14: a unique regulator for pluripotency and epigenetic reprogramming. Trends in biochemical sciences. 10.1016/j.tibs.2014.04.003 24811060

[20] Pinheiro I, Margueron R, Shukeir N, Eisold M, Fritzsch C, et al (2012) Prdm3 and Prdm16 are H3K9me1 methyltransferases required for mammalian heterochromatin integrity. Cell 150: 948–960. 22939622 10.1016/j.cell.2012.06.048

[21] Harms MJ, Ishibashi J, Wang W, Lim HW, Goyama S, et al (2014) Prdm16 is required for the maintenance of brown adipocyte identity and function in adult mice. Cell Metab 19: 593–604. 24703692 10.1016/j.cmet.2014.03.007 PMC4012340

[22] Fog CK, Galli GG, Lund AH (2012) PRDM proteins: important players in differentiation and disease. BioEssays: news and reviews in molecular, cellular and developmental biology 34: 50–60. 10.1002/bies.201100107 22028065

[23] Morishita K (2007) Leukemogenesis of the EVI1/MEL1 gene family. International journal of hematology 85: 279–286. 17483069 10.1532/IJH97.06174

[24] Schneider R, Bannister AJ, Kouzarides T (2002) Unsafe SETs: histone lysine methyltransferases and cancer. Trends in biochemical sciences 27: 396–402. 12151224 10.1016/s0968-0004(02)02141-2

[25] Kinameri E, Inoue T, Aruga J, Imayoshi I, Kageyama R, et al (2008) Prdm proto-oncogene transcription factor family expression and interaction with the Notch-Hes pathway in mouse neurogenesis. PloS one 3: e3859. 19050759 10.1371/journal.pone.0003859 PMC2585159

[26] Qian C, Zhou MM (2006) SET domain protein lysine methyltransferases: Structure, specificity and catalysis. Cellular and molecular life sciences: CMLS 63: 2755–2763. 17013555 10.1007/s00018-006-6274-5 PMC11136235

[27] Kim KC, Geng L, Huang S (2003) Inactivation of a histone methyltransferase by mutations in human cancers. Cancer research 63: 7619–7623. 14633678

[28] Derunes C, Briknarova K, Geng L, Li S, Gessner CR, et al (2005) Characterization of the PR domain of RIZ1 histone methyltransferase. Biochemical and biophysical research communications 333: 925–934. 15964548 10.1016/j.bbrc.2005.05.190

[29] Wu Y, Ferguson JE 3rd, Wang H, Kelley R, Ren R, et al (2008) PRDM6 is enriched in vascular precursors during development and inhibits endothelial cell proliferation, survival, and differentiation. Journal of molecular and cellular cardiology 44: 47–58. 17662997 10.1016/j.yjmcc.2007.06.008 PMC2683064

[30] Eom GH, Kim K, Kim SM, Kee HJ, Kim JY, et al (2009) Histone methyltransferase PRDM8 regulates mouse testis steroidogenesis. Biochemical and biophysical research communications 388: 131–136. 19646955 10.1016/j.bbrc.2009.07.134

[31] Hayashi K, Yoshida K, Matsui Y (2005) A histone H3 methyltransferase controls epigenetic events required for meiotic prophase. Nature 438: 374–378. 16292313 10.1038/nature04112

[32] Guntert P (2004) Automated NMR structure calculation with CYANA. Methods in molecular biology 278: 353–378. 15318003 10.1385/1-59259-809-9:353

[33] Nederveen AJ, Doreleijers JF, Vranken W, Miller Z, Spronk CA, et al (2005) RECOORD: a recalculated coordinate database of 500+ proteins from the PDB using restraints from the BioMagResBank. Proteins 59: 662–672. 15822098 10.1002/prot.20408

[34] Xiao B, Wilson JR, Gamblin SJ (2003b) SET domains and histone methylation. Curr Opin Struct Biol 13: 699–705. 14675547 10.1016/j.sbi.2003.10.003

[35] Xiao B, Jing C, Wilson JR, Walker PA, Vasisht N, et al (2003a) Structure and catalytic mechanism of the human histone methyltransferase SET7/9. Nature 421: 652–656. 12540855 10.1038/nature01378

[36] Zhang X, Yang Z, Khan SI, Horton JR, Tamaru H, et al (2003) Structural basis for the product specificity of histone lysine methyltransferases. Molecular cell 12: 177–185. 12887903 10.1016/s1097-2765(03)00224-7 PMC2713655

[37] Briknarova K, Zhou X, Satterthwait A, Hoyt DW, Ely KR, et al (2008) Structural studies of the SET domain from RIZ1 tumor suppressor. Biochemical and biophysical research communications 366: 807–813. 18082620 10.1016/j.bbrc.2007.12.034

[38] Wu H, Min J, Lunin VV, Antoshenko T, Dombrovski L, et al (2010) Structural biology of human H3K9 methyltransferases. PloS one 5: e8570. 20084102 10.1371/journal.pone.0008570 PMC2797608

[39] Couture JF, Collazo E, Brunzelle JS, Trievel RC (2005) Structural and functional analysis of SET8, a histone H4 Lys-20 methyltransferase. Genes & development 19: 1455–1465. 15933070 10.1101/gad.1318405 PMC1151662

[40] Jacobs SA, Harp JM, Devarakonda S, Kim Y, Rastinejad F, et al (2002) The active site of the SET domain is constructed on a knot. Nat Struct Biol 9: 833–838. 12389038 10.1038/nsb861

[41] Wu H, Mathioudakis N, Diagouraga B, Dong A, Dombrovski L, et al (2013) Molecular basis for the regulation of the H3K4 methyltransferase activity of PRDM9. Cell reports 5: 13–20. 24095733 10.1016/j.celrep.2013.08.035

[42] Kwan AH, Czolij R, Mackay JP, Crossley M (2003) Pentaprobe: a comprehensive sequence for the one-step detection of DNA-binding activities. Nucleic acids research 31: e124. 14530457 10.1093/nar/gng124 PMC219492

[43] Huang S, Shao G, Liu L (1998) The PR domain of the Rb-binding zinc finger protein RIZ1 is a protein binding interface and is related to the SET domain functioning in chromatin-mediated gene expression. J Biol Chem 273: 15933–15939. 9632640 10.1074/jbc.273.26.15933

[44] Johnson LM, Du J, Hale CJ, Bischof S, Feng S, et al (2014) SRA- and SET-domain-containing proteins link RNA polymerase V occupancy to DNA methylation. Nature 507: 124–128. 24463519 10.1038/nature12931 PMC3963826

[45] Simpson RJ, Yi Lee SH, Bartle N, Sum EY, Visvader JE, et al (2004) A classic zinc finger from friend of GATA mediates an interaction with the coiled-coil of transforming acidic coiled-coil 3. The Journal of biological chemistry 279: 39789–39797. 15234987 10.1074/jbc.M404130200

[46] Cai M, Huang Y, Sakaguchi K, Clore GM, Gronenborn AM, et al (1998) An efficient and cost-effective isotope labeling protocol for proteins expressed in *Escherichia coli* . J Biomol NMR 11: 97–102. 9566315 10.1023/a:1008222131470

[47] Shen Y, Delaglio F, Cornilescu G, Bax A (2009) TALOS+: a hybrid method for predicting protein backbone torsion angles from NMR chemical shifts. Journal of biomolecular NMR 44: 213–223. 19548092 10.1007/s10858-009-9333-z PMC2726990

[48] Laskowski RA, Rullmannn JA, MacArthur MW, Kaptein R, Thornton JM (1996) AQUA and PROCHECK-NMR: programs for checking the quality of protein structures solved by NMR. J Biomol NMR 8: 477–486. 9008363 10.1007/BF00228148

[49] Sievers F, Wilm A, Dineen D, Gibson TJ, Karplus K, et al (2011) Fast, scalable generation of high-quality protein multiple sequence alignments using Clustal Omega. Molecular systems biology 7: 539. 21988835 10.1038/msb.2011.75 PMC3261699

[50] Bond CS, Schuttelkopf AW (2009) ALINE: a WYSIWYG protein-sequence alignment editor for publication-quality alignments. Acta crystallographica Section D, Biological crystallography 65: 510–512. 19390156 10.1107/S0907444909007835

